# The impact of gender-exclusive language on women’s anticipated ostracism: A preregistered replication of Stout and Dasgupta (2011)

**DOI:** 10.1371/journal.pone.0290709

**Published:** 2023-09-20

**Authors:** Erika J. Rosenberger, Heather M. Claypool

**Affiliations:** Department of Psychology, Miami University, Oxford, Ohio, United States of America; University of Connecticut, UNITED STATES

## Abstract

In a 2011 study, Stout and Dasgupta exposed men and women to what they termed gender-inclusive language, which used both male and female referents, or to what they termed gender-exclusive language, which used male referents only. They found that, in comparison to gender-inclusive language, a job description that used gender-exclusive language negatively impacted women; they reported higher anticipated job-based ostracism and perceived sexism and lower job-based motivation and identification. This work reports a high-powered, preregistered study with women that fully replicated Stout and Dasgupta’s findings. Moreover, in an exploratory analysis, we found that, for women, gender-exclusive language is perceived as sexist, which in turn predicted feelings of greater anticipated ostracism, which in turn predicted lower job-based motivation and identification. Therefore, our findings support past research that subtle linguistic cues can be interpreted as exclusionary, that this interpretation can trigger negative outcomes, and that people can experience group-level ostracism based on their social identity.

## Introduction

While contemplating a change of employment, a woman browses a career website and encounters this in a job ad: “An effective employee has many qualities. He has effective time management, a strong work ethic, and a commitment to quality customer service. We aim to hire people like him.” How will she likely react? Will she feel encouraged to apply? Will she anticipate being welcomed at this company?

In the quoted passage, masculine pronouns (he, him) are used generically, as an alleged stand-in for those of any gender identity. However, prior work suggests that people likely interpret such language as referring to men only (e.g., [1]). Thus, for the woman job-hunter, this language may be perceived as excluding or ignoring her. Indeed, Stout and Dasgupta [2] showed that job descriptions that used masculine referents only, as in the hypothetical passage above, were interpreted by women as highly sexist and exclusionary and deflated their motivation to pursue that job and their anticipated identification with that job. The goal of the present study was to conduct a high-powered, preregistered replication of these primary findings.

### Language as a source of exclusion

Historically, scholars have used terms like “generic masculine” or “generic he” to refer to the use of male pronouns or male referents as applying to all people, regardless of their gender identity. In this work, we will use the term “gender-exclusive language” to refer to this same situation because it is the term used by Stout and Dasgupta [2] themselves (p. 757). Similarly, we will use Stout and Dasgupta’s [2] (p. 759) term “gender-inclusive language,” which has also been referred to as “gender-fair” [3], to refer to language that uses both masculine and feminine terms (e.g., he or she). To understand why women might react adversely to gender-exclusive language, it is critical to understand the importance of social belonging for human functioning and humans’ sensitivity to belonging-threats.

In their comprehensive review, Baumeister and Leary [4] argued that humans have a profound need to belong; a need to feel positively and meaningfully connected to others. They further alleged that this need stems from evolutionary pressures because being a member of a cooperative group would have been adaptive and highly beneficial for early-day humans, who would have struggled to live and thrive on their own. As such, modern-day humans have this need and are therefore highly sensitive to, and negatively impacted by, belonging-threats, which come from experiences such as loneliness, social ostracism, exclusion, and rejection (e.g., [5]). Indeed, in the wake of belonging-threats, people suffer a host of deleterious outcomes. For instance, they report decreases in their self-esteem, feelings of control, and sense of meaningful existence (e.g., [6]), have difficulties with self-regulation (e.g., [7]), have altered physical pain sensitivity (e.g., [8, 9]), show deficits in higher-order cognition [10], have altered time perception [11], and even suffer greater mortality (e.g., [12]).

Because belonging-threats are so harmful, Williams [5, 13] has argued that people should be highly sensitive to signs of them. The faster people notice their own possible ostracism, for example, the faster they can take corrective actions to protect or restore belonging, such as apologizing for their misdeeds to get back into a group or seeking belonging from another (hopefully welcoming) group (e.g., [13]). This reasoning explains a host of findings illustrating that people react quickly and strongly to even very subtle signs of any possible belonging-theat. For instance, not receiving consistent virtual ball-tosses in a meaningless game with complete strangers reliably triggers reported reductions in one’s feelings of belonging, self-esteem, control, and meaningful existence [14], and receipt of averted eye gaze is enough to thwart belonging [15].

In the examples just described, people reacted to cues of interpersonal belonging-threat, wherein they, as an individual person, were being left out, ignored, or ostracized. They personally did not receive a ball-toss or direct eye contact. But, at other times, people might experience group-level belonging-threat, wherein their social ingroup is being left out, ignored, or ostracized, even if they as individuals are not. Given the adaptive value of reacting quickly and strongly to even subtle signs of interpersonal belonging-threat, it stands to reason that people might react similarly to group-level belonging-threats.

Moreover, memberships in social groups represent key aspects of our social identities, which themselves are key aspects of the self (e.g., [16]). Thus, when our groups or identities are seemingly left out, it may threaten the self and be experienced quite similarly to direct, interpersonal belonging-threat. Work on social identity threat confirms such speculations. For instance, exposure to objects seen as highly stereotypic of computer science, which themselves seem male-stereotypic, reduce women’s interest in computer science and sense of belonging in associated computer science environments [17]. In this case, nothing was excluding an individual woman from the field. Yet, women interpreted the environmental cues as suggesting that a group of which they are a member may not belong in that space.

Similarly, linguistic cues that appear to ignore some groups could be interpreted as exclusionary by those ignored groups. Of primary relevance to the current work is the use of language that references men only while leaving out people of other gender identities. Use of generic masculine, including generic masculine pronouns (e.g., [1]), has been shown in multiple studies to be interpreted as referring mostly or primarily to men and to conjure male imagery, an effect observed across several languages including English, Polish, German, French, and others (see [18] for a brief review). If indeed male-specific referents (like he, him) are interpreted as referencing men only, and not as referencing everyone, then it stands to reason that women might feel excluded by such language, as Stout and Dasgupta [2] found.

### Replicating Stout and Dasgupta (2011)

As noted above, the goal of this work is to investigate the primary findings from Stout and Dasgupta [2]; that women feel excluded by gender-exclusive language, which also hampered downstream consequences on job motivation and identification. Accordingly, it is important to delineate exactly what Stout and Dasgupta [2] did and found in their studies, as well as note why we think replicating their primary findings is important.

They conducted three experiments to test their hypotheses. In their first, college students (analyzed *N* = 164; 92 women and 72 men) read a job description that they were to evaluate while imagining they were searching for a job post-graduation. In the “gender-exclusive condition,” the job description included only masculine pronouns and terms (he, him, guys), whereas in the “gender-inclusive condition,” the description used both masculine and feminine pronouns and gender-neutral terms (e.g., he or she, him or her, employees; [2], p. 760). After reading the description, participants completed measures of anticipated job-based ostracism, perceived sexism, and job-based motivation and identification. Anticipated job-based ostracism captured how much the participants expected to be ignored at the job if they were to work there. Perceived sexism measured how much the participants believed the job description favored men or women. Job-based motivation captured how motivated the participants would be in the employment setting, and job-based identification measured how central the job would be to their self-concept. Stout and Dasgupta discovered that both men and women perceived the gender-exclusive job (compared to the gender-inclusive one) as more sexist. But, only women anticipated greater ostracism, lower job-based motivation, and lower job-based identification in the gender-exclusive (compared to the gender-inclusive) condition. On these three measures, men did not differ across language conditions, with one exception: they reported *greater* job-based motivation in the gender-exclusive (versus inclusive) condition.

In their Study 2, Stout and Dasgupta [2] aimed to replicate the Study 1 findings in a mock interview setting. Here, a male interviewer used either gender-exclusive, gender-inclusive, or gender-neutral language when describing the alleged job. The latter condition, new to this study, did not use gendered pronouns at all, but instead used terms like “employee” to refer to individual workers. After hearing their assigned version of the alleged job, participants (analyzed *N* = 248; 149 women, 99 men) again completed measures of perceived sexism, job-based motivation, and job-based identification as well as a “sense of belonging in the workplace” ([2], p. 762). This latter variable was conceptually a reverse-coded version of the anticipated ostracism measure from Study 1. Compared to the gender-inclusive and gender-neutral conditions (which did not differ from each other), women in the gender-exclusive condition reported higher perceived sexism and lower sense of belonging, motivation, and identification. Men found the gender-exclusive job description more sexist than the gender-inclusive or gender-neutral versions, but men did not report differences on any of the other dependent measures as a function of language condition. Thus, this study expanded on the findings of Study 1 in two ways. First, it showed that women react adversely to gender-exclusive language when encountering it in an out-loud, oral context (Study 2), as well as in written form (Study 1). Second, it showed that gender-exclusive language is harmful to women, but gender-inclusive language impacts women the same as gender-neutral language.

Study 3 was procedurally very similar to Study 2, except it used women only as participants (analyzed *N* = 88), it assessed women’s feelings of belonging *during* the interview (instead of their expectations of belonging in the future job), and women were unobtrusively video recorded during the interview so that judges could later code their nonverbal emotions. As predicted, women in the gender-exclusive-language condition (compared to the other two) found the language used more sexist, displayed more negative nonverbal reactions as the interview progressed, and reported less belonging during the interview, as well as lower job-based motivation and job-based identification. Overall, then, these results again confirmed that, compared to gender-neutral and gender-inclusive language, exposure to gender-exclusive language is harmful to women.

The current study’s aim is to conduct a high-powered, preregistered replication of Stout and Dasgupta’s [2] Study 1, focused on women only, since only women experienced psychologically adverse reactions to the gender-exclusive language. These are important findings to replicate for three reasons. First, these findings have important applied and theoretical implications; therefore, replicating them would add greater empirical evidence to bolster these implications. On the theoretical level, much past research in the belonging-threat literature has focused on these threats at the individual level. But, Stout and Dasgupta’s findings illustrated that people can experience belonging-threats when their social ingroups, even if not them personally, are ignored, left out, or ostracized. On the applied level, Stout and Dasgupta’s findings showed that encountering gender-exclusive language in a potential employment setting has substantial costs for women. If Stout and Dasgupta’s findings [2] are correct, then women encountering this language may anticipate rejection in that potential job setting and, therefore, may opt not to accept that job if offered or apply at all, as withdrawing from these sorts of situations is a common reaction to perceived rejection (e.g., [19]). If job descriptions, policies, and other professional settings use gender-exclusive language, it might prompt women to avoid or withdraw from valuable work opportunities, exacerbating workplace gender disparities (see [18]).

Second, the original studies appear underpowered. The sample sizes Stout and Dasgupta [2] used were quite normative in the field at the time, yet were likely insufficient to assure satisfactory statistical power. Based on our a priori power analysis (see methods for more details), 172 women-identifying participants (86 per language condition) would be needed to achieve 80% power to detect their smallest effect from Study 1. Their Study 1 included 92 women across both the gender-inclusive and gender-exclusive conditions (thus, only 46 women per condition). Similarly, their Study 2 included 149 women across three language conditions, with thus only ~50 per condition. And, in their Study 3, they analyzed data from 88 women across three language conditions, thus yielding ~30 participants per condition. The field is now more aware that low-powered studies tend to produce exaggerated effect sizes [20] and may also lead to higher false positive rates [21]. Therefore, it is important to examine Stout and Dasgupta’s findings with a better-powered study to gain additional confidence in the original findings and perhaps better estimates of their effect sizes.

Third, preregistration was not common practice at the time Stout and Dasgupta [2] conducted their work and, thus, not surprisingly, their work was not preregistered. Yet, preregistration enhances the credibility of research findings by clarifying which components of the study were planned and which were exploratory [22]. Thus, by preregistering our hypotheses and planned methods and analyses, confidence in our findings, as well as theirs, can be augmented (see here for the preregistration: https://osf.io/g2svd).

### Current research

This work sought to replicate, in a high-powered and preregistered study, the primary findings from women from Stout and Dasgupta’s [2] first study. Namely, women-identified participants read a job description that used either gender-exclusive (e.g., he/him) or gender-inclusive (e.g., he or she/him or her) language. We preregistered our expectation that we would replicate the findings of Stout and Dasgupta [2]. Namely, we predicted that women who read the gender-exclusive job description (compared to the gender-inclusive one) would report (1) greater feelings of anticipated ostracism, (2) greater perceived sexism, (3) lower feelings of job-based motivation, and (4) lower job-based identification.

## Methods

### Participants, sample size rationale, and data-collection stopping rules

We conducted an a priori G*Power analysis (using version 3.0.10, [23]) to determine the needed sample size to achieve 80% power. We based our anticipated effect size on the findings reported in Study 1 of Stout and Dasgupta [2]. In it, the authors reported effect sizes representing the difference between women in the gender-exclusive and women in the gender-inclusive language conditions on their key dependent variables. The smallest of these effect sizes from their Study 1 was *d* = .43, observed on the job-based identification dependent variable. With an assumed effect size of *d* = .43, G*Power calculated that we needed 172 women-identifying participants (86 per language condition) to achieve 80% power (see the preregistration for more details; https://osf.io/g2svd).

To acquire 172 usable participants, we planned to recruit more than this because we preregistered our intentions to analyze data only from those who both identified as women and passed the attention check (see methods below for more details). Put differently, because we anticipated having to omit some participants before performing the primary analyses, we targeted for recruitment a number greater than 172.

In total, we recruited 191 participants, deviating slightly from the preregistered target of 190, due to a standard “overbooking” scheduling process in the lab to account for possible “no-show” participants. These participants completed the study for college credit. Per our preregistration, at this stage, we examined the responses to the attention-check and the gender-identification demographic questions only to determine how many participants, if any, needed to be removed from the dataset before primary analyses began. Three of the 191 participants did not identify as a woman, and an additional 12 participants failed the embedded attention check. This left a final, analyzable dataset of *N* = 176. Because this value surpassed our desired sample size (of 172), data collection stopped at this point, as planned in the preregistration. Of the participants included in the analyses, 85.8% of them identified as Caucasian/White, 5.7% identified as Asian American/Asian, 3.4% identified as African American/Black, 3.4% identified as multiracial, 0.6% (one participant) chose to self-describe their race (as “Middle Eastern”), and 1.1% did not report their race. Additionally, participants (regardless of their racial-identification) were asked to indicate if they did or did not identify as Latino/a/x, and 4.0% did.

### Procedure

The Psychology Departmental Review Board subcommittee of the Miami University Institutional Review Board approved this protocol as meeting all ethical guidelines (approval #02021r). Adult participants (aged 18 or older) signed up for a study allegedly about “evaluating job materials” and reported to the lab. They were handed a written consent form by the research assistant before the study started, and this assistant was available to answer any questions about the form if they arose. All participants read and signed the form to indicate their consent to participate. Each participant was then seated at a computer station inside a private room, and the trained research assistant launched the study, which was hosted on the Qualtrics platform. Once the study began, participants were instructed (as they were in Stout and Dasgupta [2], Study 1) to imagine that they were looking for a job after graduation and, with this in mind, to read a description of a company with a job opening and consider how they would feel applying and working there.

Based on random assignment, participants then read one of two company/job descriptions, with each representing one of the two language conditions. In the gender-exclusive condition, masculine referents and terms (he, him, guys) were used, whereas in the gender-inclusive condition, masculine and feminine referents and gender-neutral terms were used (e.g., he or she, him or her, employees). These descriptions were taken verbatim from Stout and Dasgupta’s [2] Study 1 (see their Appendix for exact wording).

After reading the assigned description, participants answered questions concerning their perceptions of the job/company, which assessed the same four key dependent variables used by Stout and Dasgupta: anticipated job-based ostracism, perceived sexism, job-based motivation, and job-based identification. Stout and Dasgupta [2] reported using two items to measure anticipated job-based ostracism, four items to measure motivation, four items to measure identification, and three items to measure perceived sexism. These authors provided verbatim wording in the article for both their job-based ostracism items but listed only two of four items each for motivation and identification and only one of the three items for perceived sexism. In our replication study, we used all the verbatim-provided questions and thus fewer items to assess three of these four variables than did Stout and Dasgupta. Moreover, for perceived sexism, we created an item to avoid using a one-item measure. Thus, all items described next were taken verbatim from those reported in-text in Stout and Dasgupta’s [2] Study 1, with one exception.

Namely, anticipated job-based ostracism was measured using the two Stout and Dasgupta [2] (p. 760) items: “To what extent do you feel that you would be ignored or excluded by your colleagues?” and “To what extent do you feel that you would be noticed or included by your colleagues?” Both items were rated on a 7-point Likert scale from *Not at all* (1) to *Very much so* (7). The second item was reverse scored and then averaged together with the first item to form an anticipated job-based ostracism index (ρ = .887).

Perceived sexism was measured using Stout and Dasgupta’s [2] (p. 760) item, “Do you think that the writing style in the job description favored one gender over the other?” and our self-written item, “Do you think the job description was addressed to one gender over the other?” Both items were rated on a scale of *Favored women* (1) to *Favored men* (7). A perceived sexism index was created by averaging the items together (ρ = .922).

Job-based motivation was measured using the two Stout and Dasgupta [2] (p. 760) items, “How motivated do you think that you would be in this work environment?” and “How likely would you be to think about your work outside of work hours because you want to, not because you are expected to?” Participants responded to the former item on a *Not at all motivated* (1) to *Very motivated* (7) scale and responded to the latter on a *Not at all likely* (1) to *Very likely* (7) scale. The job-based motivation index was created by averaging the two items together (ρ = .572).

Job-based identification was measured using the two Stout and Dasgupta [2] (p. 760) items, “How important would this job be to your self-concept?” and “How much personal satisfaction would you get out of your work if you were working in this environment?” Participants responded to the former item on a *Not at all important* (1) to *Very important* (7) scale and responded to the latter on a *No satisfaction* (1) to *A great deal of satisfaction* (7) scale. The items were averaged together to create the job-based identification index (ρ = .655).

The just-described eight items were presented in a randomized order, and embedded among them was one attention check item, “Please select a value of 3 to indicate that you are paying attention,” and participants replied on a *Not at all* (1) to *Very much so* (7) scale. Per the preregistration plan, participants who responded with a score of 3 “passed” the check, and participants who left this question blank or replied with any value other than 3 “failed” the check. Those failing the check were omitted from the dataset before the primary analyses were conducted.

To conclude the study, participants answered a few demographic questions and then were thanked, debriefed, and dismissed from the lab.

## Results

### Preregistered analyses

Stout and Dasgupta [2] found that women in the gender-exclusive condition (compared to the gender-inclusive condition) anticipated more job-based ostracism, perceived the job/company description to be more sexist, and had lower job-based identification and job-based motivation. We preregistered our expectation to replicate these exact findings. To test these hypotheses, we conducted preregistered independent samples *t*-tests, comparing those in the gender-exclusive condition to those in the gender-inclusive condition, separately for each outcome measure. The obtained results fully replicated those of Stout and Dasgupta [2]. As expected, women in the gender-exclusive condition reported significantly greater anticipated job-based ostracism, significantly less job-based motivation, significantly less job-based identification, and significantly stronger beliefs that the job/company description was sexist, compared to women in the gender-inclusive condition. See Table 1 for all means, standard deviations, effect sizes, and associated inferential statistics. See here (https://osf.io/uxc58) to access the dataset itself.

**Table 1:** Descriptive and inferential statistics as a function of language condition.

Measure	Gender-Exclusive Mean (*SD*)	Gender-Inclusive Mean (*SD*)	*df*	*t*	*p*	*d*
**Job-based ostracism**	5.08 (1.61)	3.34 (1.37)	174	7.71	< .001	1.16
**Perceived sexism** ^*^	6.15 (1.28)	3.89 (.93)	158.83	13.42	< .001	2.02
**Job-based motivation** ^*^	3.63 (1.56)	4.65 (1.19)	162.75	-4.86	< .001	-0.73
**Job-based identification** ^*^	3.31 (1.39)	4.71 (1.07)	163.23	-7.47	< .001	-1.13

For measures marked with an

*, the Levene’s Test for Equality of Variances was significant. Thus, for these, we report in the table the *df*, *t*-value, and *p*-value for the associated *t*-test that does not assume equal variances.

### Exploratory analyses (not preregistered)

Having unambiguously replicated the key findings from Stout and Dasgupta’s [2] Study 1, we next conducted an exploratory analysis to investigate possible mediational processes among the manipulated and measured variables. Namely, Stout and Dasgupta’s [2] theoretical framework argued that “women and men alike would perceive gender-exclusive language as more sexist than nonexclusive language but only women would be personally affected by it. Specifically, gender-exclusive language would deflate women’s sense of belonging, lower their motivation to purse the job, [and] produce disidentification with the job” (p. 759). This framing suggests that gender-exclusive language should psychologically trigger perceptions of sexism first among women, which signals to them that they do not belong in that sexist context. This, in turn, should make women wish to withdraw from and care less about that context. Put differently, our reading of Stout and Dasgupta’s framing suggests that the impact of the language manipulation on job-based motivation and identification should be serially mediated by perceived sexism and anticipated ostracism, in that order, a novel analysis that Stout and Dasgupta did not report.

**Fig 1:**
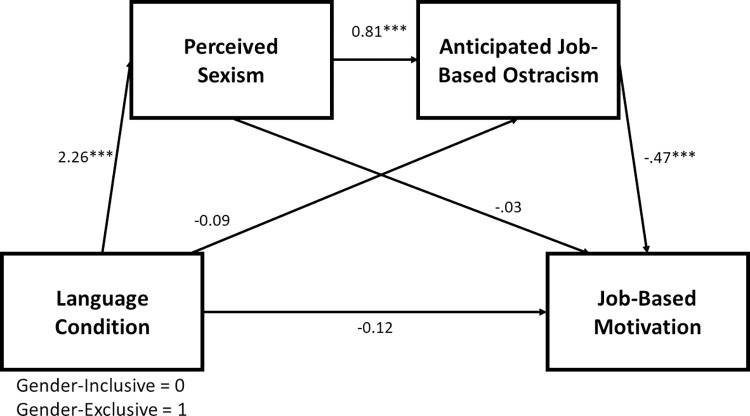
Serial mediation from language condition to job-based motivation. The impact of language condition on motivation was serially mediated by perceived sexism and anticipated ostracism, Indirect Effect = -.86, *SE* = .18, 95% CI = [-1.23, -.53]. *** *p* < .001.

**Fig 2:**
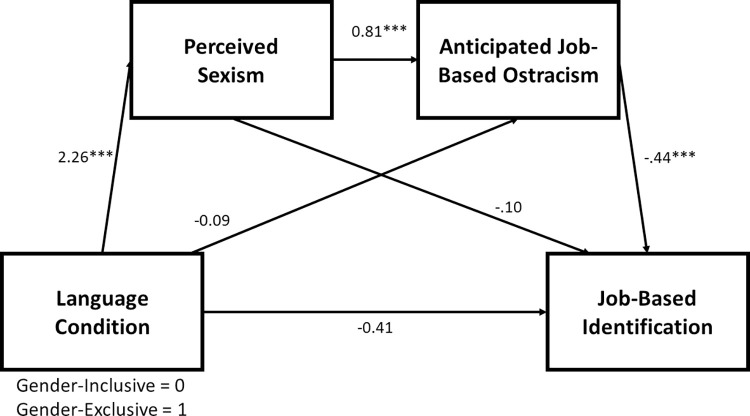
Serial mediation from language condition to job-based identification. The impact of language condition on identification with the job was serially mediated by perceived sexism and anticipated ostracism, Indirect Effect = -.80, *SE* = .16, 95% CI = [-1.13, -.51]. *** *p* < .001.

Thus, we ran a pair of mediational analyses to test this exploratory serial mediation model. We ran Hayes’s [24] PROCESS (version 4.1) Model 6 with 10,000 bootstrapped samples and seed = 125, requesting 95% confidence intervals for the indirect and direct effects. In the first of these models, language condition (0 = gender-inclusive; 1 = gender-exclusive) served as the manipulated independent variable, job-based motivation served as the outcome/dependent variable, and perceived sexism and anticipated job-based ostracism served as potential serial mediators (in that order). The second run of this model was identical, except job-based identification was the outcome/dependent variable. Consistent with our exploratory hypothesis, the serial indirect effect of language condition on job-based motivation via perceived sexism and anticipated ostracism was significant as was this same serial indirect effect on job-based identification. See Figs 1 and 2. Thus, the results from these exploratory analyses offer initial support for the conclusion that gender-exclusive language is perceived as sexist by women, which predicts feeling more ostracized, which predicts greater desires to withdraw psychologically from that context (via lowered job-based motivation and identification).

## Discussion

Stout and Dasgupta’s [2] Study 1 found that women who read a job description that used gender-exclusive (e.g., he/him) compared to gender-inclusive (e.g., he or she/him or her) language were negatively impacted: they had higher expectations of ostracism, perceived the job description as more sexist, and reported lower motivation and identification with the job. Due to field-level norms when the study was conducted, that work was likely underpowered, and it had not been preregistered. We attempted to replicate these findings in a high-powered and preregistered study to potentially support the prior evidence.

Our results, indeed, fully replicated the primary findings of Stout and Dasgupta’s [2] Study 1. Namely, we observed that women in the gender-exclusive condition had (1) higher anticipated job-based ostracism, (2) higher perceived sexism, (3) lower job-based motivation, and (4) lower job-based identification than women in the gender-inclusive condition. Not only were the results statistically significant (all *p*s < .001 for these group-difference comparisons), but the effect sizes capturing the language-condition differences were quite large for all four outcomes (|*d*s| > 0.72). Therefore, the current findings unambiguously support the original conclusion from Stout and Dasgupta [2] that gender-exclusive language has deleterious effects on women.

Replicating Stout and Dasgupta’s [2] findings with a high-powered and preregistered study increases the confidence in the results and provides a better estimate of the effect sizes. Moreover, these results support past research that people are attuned to subtle indications of belonging-threat (e.g., [13]), including group-level threats that target one’s social identity, which complements the social identity threat literature. Just as people notice visual cues (e.g., *Star Trek* posters; [17]), people notice linguistic cues that they interpret as exclusionary, and various negative consequences follow.

In addition to replicating Stout and Dasgupta’s prior findings, we extended them in a novel way. Specifically, we conducted exploratory (non-preregistered) process analyses based on the theoretical framework described by Stout and Dasgupta [2]. In doing so, we found preliminary evidence consistent with a causal model, wherein perceived sexism and anticipated ostracism acted as serial mediators (in that order) between language condition and the outcome variables (motivation and identification). This finding suggests that women perceived the gender-exclusive language to be sexist, which predicted greater expectations of being ostracized at the job. This reduced sense of belonging predicted withdrawal in the forms of reduced motivation and identification. Of course, though this mediational analysis provides evidence consistent with this model, additional theoretical and empirical work is needed to establish whether this exploratory causal chain is viable (e.g., [25]).

Practically, Stout and Dasgupta’s [2] and our current findings demonstrate the importance of carefully considering the language used in high-impact, high-stakes situations, such as job recruitment. Indeed, gender-exclusive language could explain, in part, gender-based hiring disparaties and other gender inequities. When generic masculine pronouns are used in these contexts, women feel that they will not belong, as shown by Stout and Dasgupta [2] and our current findings. Other forms of gender-exclusive language may also produce similar results. For example, when masculine (e.g., “competitive”) as opposed to feminine (e.g., “interpersonal”) wording that elicits gender stereotypes was used for job recruitment, women reported less interest in the job due to a lower sense of anticipated belonging ([26]; see [27] for similar findings). Not only do women withdraw themselves, but others’ perceptions of women can be influenced by gender-exclusive language, too. For instance, Horvath and Sczesny [28] found that when masculine forms of words were used in job advertisements, women applicants were perceived as less fit than men for a CEO position.

It is evident from our results and those from related work that more inclusive forms of language should be used in settings with lasting societal consequences. Gender-inclusive types of language, broadly defined, can be created by using both male and female pronouns, as was the case in the current work, or by using *neutralization*, wherein one replaces gendered language with non-gendered forms [3]. For example, changing “master” to “head” eliminated cognitive biases favoring men over women [29]. Another route to more inclusive language is via *feminization*, which presents both masculine and feminine forms of a word (e.g., waiters and waitresses; see [3] for a review). For instance, presenting children with professions in pair form instead of just the generic masculine form has been shown to increase girls’ interest in those professions [30]. Additionally, using masculine-feminine word pairs for professions has been found to increase women’s visibility [31].

There are advantages and disadvantages to both methods. On one hand, neutralization may reduce the salience of gender but may yet still be influenced by gender stereotypes (e.g., “firefighter” still results in male bias; [32]). On the other hand, feminization increases women’s visibility; however, it does not include non-binary individuals, and some feminine terms and titles are devalued (e.g., [33, 34]). Therefore, it is important to consider the context and one’s objectives when deciding on and implementing a specific gender-inclusive strategy (see [35] for a review). Nevertheless, the current work adds to the growing evidence that gender-exclusive language is damaging to women and that deliberate efforts should be putforth by leaders to assuage this damage [3, 35].

### Limitations

As with all research, this study has limitations. First, we analyzed data from only women participants instead of both men and women like Stout and Dasgupta’s [2] study. Importantly, Stout and Dasgupta [2] found that only their women participants were negatively impacted by written (Study 1) or spoken (Study 2) gender-exclusive language; though men agreed that gender-exclusive language was sexist, they did not anticipate ostracism or report reduced motivation or identification as a result of the sexist language. Therefore, though some comparison information was lost by not examining men, we believe it was logical and justifiable to recruit only women for this replication study. Nevertheless, this limitation is worth noting.

Second, we did not include a condition that only used gender-neutral terms (e.g., “one”). Without a gender-neutral control, it is difficult to determine conclusively if gender-exclusive language is harmful for women, whether gender-inclusive language is helpful for women, or both. However, Stout and Dasgupta’s [2] Studies 2 and 3 (which both had a gender-neutral condition) revealed that the gender-neutral and gender-inclusive conditions did not differ from one another, and themselves concluded that gender-exclusive language is harmful for women, but gender-inclusive language provides no benefits to women beyond gender-neutral language. Thus, our decision to omit a gender-neutral condition likely did not cost us any novel information, but again, is a limitation worth noting.

Third, we targeted just 80% power. Well-conducted replications should have high power, and some suggest that though 80% is acceptable, even higher power is desirable (e.g., [36]). If a replication is underpowered and does not reproduce the original findings, readers wonder if the original finding was a false positive or if it simply was not observed due to low power. Fortunately, in the current work, the original findings were reproduced and with large effect sizes. Thus, though targeting only 80% power might be a limitation of this work, it did not lead to the aforementioned interpretational ambiguity in this case.

Fourth, it is feasible that our large effect sizes were the result, in part, of demand characteristics. Generic masculine in written language is relatively rare in modern contexts. So, encountering it during the study might have led some participants to guess the hypothesis under investigation and adjust their responses on the self-report measures accordingly. We do not have data to directly rule out this possibility. However, some findings from Stout and Dasgupta’s [2] Study 3 are relevant here.

As discussed in the introduction, women in this study were secretly recorded during the interview, and judges (who were unaware of the study’s hypothesis and language condition) later coded their nonverbal emotions. Relative to women in the gender-neutral and gender-inclusive conditions, women in the gender-exclusive condition showed significant decreases in their implicit/nonverbal emotions. That is, their implicit nonverbal reactions got significantly worse as they listened to the gender-exclusive language; an effect more strongly observed compared to the other two conditions. This suggests that women in the gender-exclusive language condition were truly harmed by that language, as implicit reactions are difficult to “fake,” and that these findings were not merely the result of demand. Moreover, within the gender-exclusive condition only, women’s negative changes in implicit/nonverbal emotions predicted lower self-reported motivation and identification. Because women’s nonverbal reactions in the gender-exclusive condition predicted their explicit, self-reported job-based motivation and identification, this suggests that the self-reported outcomes were genuinely experienced as well. Future work that attempts to replicate the conceptual findings from Stout and Dasgupta [2] might consider adding a more implicit measure (like judgments of nonverbal affect) to provide additional evidence against a demand-characteristic interpretation.

Lastly, our sample consisted of predominantly White college-aged students. Therefore, the results are not generalizable to other racial and age groups. With intersecting marginalized identities, it is possible that the impact of gender-exclusive language is even more harmful for women of color. Conversely, older women may be more accustomed to gender-exclusive language because it has been normative through much of their lifespan, and thus, these women might be less negatively influenced. A more diverse sample in future research could investigate these possibilities.

### Future directions

There remains much to explore regarding gender and linguistic ostracism. For example, as Stout and Dasgupta [2] pondered, how might men react to generic *feminine* language? Generic feminine is a form of gender-exclusive language, in that it uses feminine referents only to refer to people of all gender identities. If men encountered a job advertisement with generic feminine language, would they perceive it as sexist and anticipate ostracism at the job? Would such perceptions lead to negative downstream consequences, like reduced motivation and identification? Because men have been and continue to be an advantaged, privileged group, perhaps unaccustomed to linguistic marginalization, they might perceive generic feminine as even more aversive than women’s response to generic masculine.

Furthermore, the general public’s understanding of gender identities has changed and expanded since the publication of Stout and Dasgupta’s work, now over a decade ago. He/him and she/her are not the only pronouns people use. For instance, many non-binary individuals use singular they/them pronouns exclusively or in addition to masculine and/or feminine pronouns. Thus, what Stout and Dasgupta conceptualized as gender-inclusive (he or she/him or her) at the time of their work is likely now not considered fully inclusive, as it is likely gender-exclusive to non-binary folks. Indeed, even in our current work, the term “gender-inclusive language” is arguably applicable only to the *participants in the study themselves* (as we analyzed only women-identifying participants), but it is likely not inclusive of all possible gender identities. Accordingly, future research should determine if non-binary individuals perceive language that uses only he or she/him or her as exclusionary and, if so, if it sparks negative downstream consequences.

As one final example, the context in which gender-exclusive language is used might influence its impact on women. For instance, many historic documents, such as the United States Constitution, include generic masculine pronouns because that was normative in its time. Such documents were also written when women had fewer rights, and therefore, the usage of this form of gender-exclusive language likely reflected women’s social status. If women encounter an historic document that uses gender-exclusive language, would they still show aversive reactions to it? Or, would such negative effects be muted, or gone entirely, because women take into account the time period in which the document was drafted? Overall, then, there are many interesting and pertinent issues to examine regarding gender-exclusive language.

## Conclusion

The language we use can have significant impacts on people by threatening their sense of belonging via their social identity. Our high-powered and preregistered study fully replicated Stout and Dasgupta’s [2] primary findings that women reported higher anticipated ostracism and perceived sexism and lower motivation and identification when a job description used gender-exclusive language (e.g., he/him) compared to gender-inclusive language (e.g., he or she/him or her). Theoretically, the current work supports past research that subtle cues, including linguistic ones, can be interpreted as a form of rejection, and people are impacted by group-level ostracism. Practically, our findings indicate that gender-exclusive language should be avoided, especially in high-stakes, consequential situations. Indeed, for our job-seeking woman referenced at the start of this paper, the current findings suggest that she would be more inclined to apply and anticipate being welcomed at the company if a more gender-inclusive version of the company was provided.
